# Supplementary material for: Mutations in PCYT2 disrupt etherlipid biosynthesis and cause a complex hereditary spastic paraplegia
Source: Brain. 2019 Oct 22;142(11):3382–97. doi: 10.1093/brain/awz291 (PMC6821184; doi:10.1093/brain/awz291)
Supplement: awz291_Supplementary_Data [file awz291_supplementary_data.zip › awz291-Suppl_data/Supplementary_Data4.zip!/Supplementary information 4 - plasma lipidomics.zip!/Plasma lipidomics/Control vs Patient3b/Statistical plots and data/PS species/Boxplots.pdf]

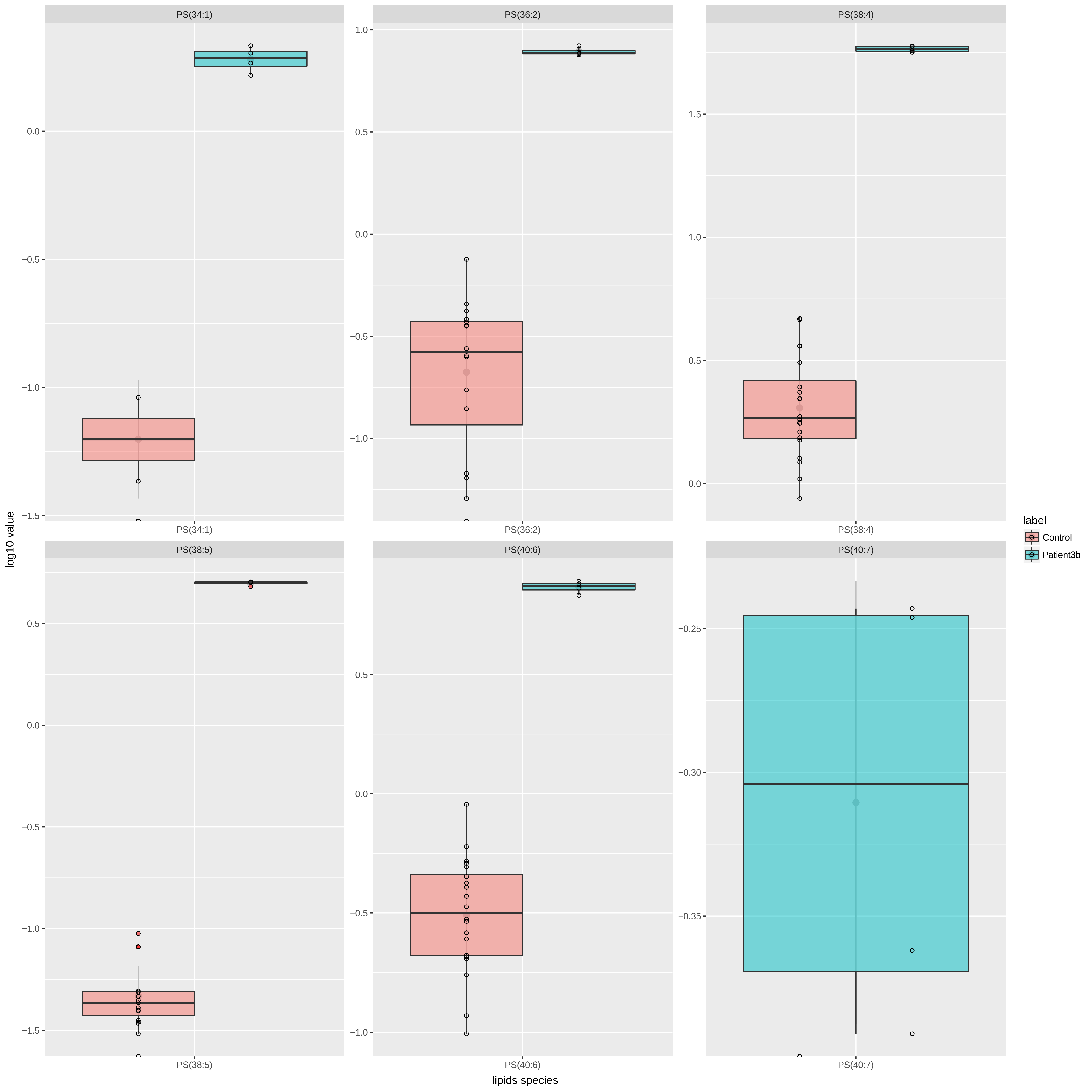

PS(34:1)
PS(36:2)
PS(38:4)
PS(38:5)
PS(40:6)
PS(40:7)
log10 value
lipids species
label
Control
Patient3b
0.0
−0.5
−1.0
−1.5
1.0
0.5
1.5
−0.25
−0.30
−0.35
